# The support group impact on enhancing the self-worth of women who terminated a pregnancy in adolescence

**DOI:** 10.4102/safp.v65i1.5707

**Published:** 2023-12-27

**Authors:** Botshelo R. Sebola, Patrone R. Risenga

**Affiliations:** 1 Department of Health Studies, School of Social Sciences, University of South Africa, Pretoria, South Africa

**Keywords:** adolescence, enhancement, self-forgiveness, self-worth, termination of pregnancy, women

## Abstract

**Background:**

Terminating a pregnancy can be a traumatic life event, resulting in negative emotions that can diminish women’s self-worth. Support from different sources, including health support groups, could be beneficial in restoring a woman’s self-worth. This article aimed to present findings on the impact of a support group intervention on the self-worth of women who terminated a pregnancy in adolescence.

**Methods:**

A qualitative, exploratory and descriptive approach, using in-depth, semi-structured interviews, was used to collect data. Thematic analysis guided the data analysis.

**Results:**

Five themes emerged from the data: reasons for joining the support group; enhanced emotional and physical wellbeing; self-forgiveness; spiritual growth and participants’ voices.

**Conclusion:**

Participants described why they needed to join the support group. It became clear that the intercession was effective in the short term as it enhanced participants’ self-worth by encouraging them to accept themselves thereby altering their self-condemning emotions, replacing them with self-love.

**Contribution:**

The study recommends that a support group should be considered as a backup for women who terminated a pregnancy and have lost their self-worth.

## Introduction

Pregnancy termination has been practised for decades, mostly for health reasons.^1^ However, there are various reasons adolescent girls fall pregnant and terminate a pregnancy. Research has determined that falling pregnant during adolescence changes the adolescent girl’s life course.^2,3^ Studies on women’s experiences of the termination of a pregnancy (TOP) are not conclusive, with some women sharing positive experiences while others experience it negatively.^4^ Termination of pregnancy studies may not necessarily be reliable as proponents downplay the negative effects, while opponents exaggerate the effects.^5^ Adolescent pregnancy termination affects individuals’ physical, emotional and spiritual wellbeing. For instance, symptoms of post-traumatic stress disorder (PTSD) have been associated with a TOP during adolescence, with one of the signs being feelings of worthlessness.^6^

Smedema, Catalano and Ebener define self-worth as the internal sense of being good enough and worthy of love, respect and belonging with others.^7^ People with a healthy self-worth have a realistic view of themselves and their abilities and believe they are worthy and valuable as people, despite not being the smartest.^8^ They do not need to compare themselves with others. This view is further supported by other scholars^9^ who claim that a strong self-worth positively impacts important life outcomes such as health and relationships. On the other hand, self-esteem is defined as what people believe they need to be or do to have value and self-worth.^10^ Scholars^11^ describe self-esteem to be the extent to which an individual evaluates themselves favourably, thus making positive self-evaluation the essential process underlying self-esteem, with people using different standards and domains to determine their esteem. Hence, self-esteem is what we think, feel and believe about ourselves^11^ in comparison to others. Both self-worth and self-esteem, are associated with wellbeing and satisfaction with life.^7^ For women who terminated a pregnancy and suffer self-punishment or self-condemnation, the expression of unconditional positive regard by the counsellor may help to improve these women’s sense of self-worth.^12^

Terminating a pregnancy can be a stressful and traumatic life event^5^ and support from different sources, including health professionals, family or support groups could be beneficial.^13^

Reliable evidence has built up over time, confirming the success of support groups for people with mental, emotional, and spiritual disturbances.^13^ A support group refers to a meeting of people with similar experiences and conditions, such as women who terminated a pregnancy, aiming to give and receive non-professional, non-clinical support and companionship from one another.^13^ The assistance is based on respect and responsibility for one another, as well as a shared agreement. Concentrated interaction among group members provides hands-on, emotional and social support in a non-threatening, no treatment-based and normalising relationship.^14^

Although some adolescents who are emotionally traumatised by TOP and have lost self-worth and may take the initiative to consult professional mental health services before their situation worsens, they are deemed the most unlikely group to seek professional mental health help.^14,15^ This phenomenon emphasises their need for one another’s support to enhance and sustain their self-worth. Observing others’ behaviours within the group can influence improvements in one’s behaviour by inspiring a belief in one’s own ability to make it.^16^

Little is written and understood about support groups and their benefits for women traumatised by a terminated pregnancy in adolescence who consequently lost their self-worth. This study intended to fill this gap.

### Aim and objectives

The purpose of this study was to explore and describe, from the women’s perspective, the impact of a support group intervention on the enhancement of self-worth among women who terminated a pregnancy in adolescence.

The following objectives were defined for the study:

- to explore and describe women’s lived experiences related to TOP.
- to evaluate a support group’s impact on the self-worth of women who terminated a pregnancy in adolescence.

### Support group intervention

#### Motivation for forming a support group

In 2018 the first researcher interviewed women who terminated a pregnancy in adolescence at a community health centre (CHC) for her doctoral study titled ‘Self-forgiveness for women who terminated pregnancy in adolescence’. Some major findings of this study were that women suffered self-condemnation and carried a burden of secrecy.^17^ The findings motivated the first researcher to encourage and oversee women who terminated a pregnancy and were lacking self-worth, to form a support group and to subsequently follow up its impact on these women through this study. As a secular intervention, the support group was formed in February 2020.

#### Recruitment of the support group

Five participants were recruited by the manager of the Family Planning clinic of the CHC, while one, who participated in the first researcher’s doctoral study, was telephonically recruited by the first researcher and one of the participants in the support group invited a woman through snowballing, as group membership was open. The researcher handed information leaflets to the clinic manager, explaining the intention of the support group, confidentiality, the running of the group, type of the support group, the meetings venue, the presentation of lessons and that interviews would be conducted after group termination. To build trust, the manager introduced each willing potential participant to the researcher, who further ensured each understood the contents of the information leaflet. The information leaflets were further explained by the first researcher to the other two, snowballed participants. All seven participants met the inclusion and exclusion criteria (see section Population and sampling) and indicated they could be addressed in English or Sesotho.

#### Activities of the support group

Two participants volunteered to co-ordinate the group activities under the researcher’s guidance. Meetings were intended to clarify feelings, reduce self-condemnation and isolation, facilitate appropriate mourning, increasing self-esteem and self-worth and bring closure to the TOP experience.^18^ Teaching methods involved lectures, discussions, questions and answers as well as sharing on emotional growth.

Participants firstly discussed and agreed upon their knowledge deficits, for example, questioning God’s forgiveness, hence the need for a presentation on divine forgiveness. Secondly external presenters were invited to address two topics, face-to-face (see Table 1). Three lectures by the 1^st^ researcher were posted and presented on telephonic group chat (see Table 1). Any follow-ups on lectures were carried out telephonically with the relevant presenter. The group was terminated in October 2021. Interviews were conducted in March 2022, after a 4 months break for reflection.

**TABLE 1:** Support group interventions.

Session	Topic	Method of presentation	Presenter
1	**Forgiveness** Relationship of God and man. Immorality: An explanation God’s forgiveness.	Lecture and discussions	A female Theologian
2	Termination of pregnancy: Positive lessons and negative outcomes	Group sharing and discussions	1st Researcher
3	**Self-forgiveness:** Guilt and shame: Differences	Mobile phone audio lecture Group discussions	1st Researcher
4	**Self-forgiveness:** Accepting accountability for aborting Sorrow	Mobile phone audio lecture Group discussions	1st Researcher
5	**Self-forgiveness:** Seeking to make amends and reaffirming personal values violated Obtaining renewed compassion, self-acceptance and self-respect	Mobile phone audio lecture Group discussions	1st Researcher
6	**Assertiveness skills** Developing self-awareness Assertiveness and aggression: differences Decision making Communication skills Personal growth Setting boundaries Coping strategies	Lecture: Face-to-face Group discussions	Psychiatry lecturer

## Methods

### Study design

A qualitative, explorative and descriptive design was used, guided by a phenomenological approach.^19^ The qualitative design was deemed appropriate because the researchers aimed to investigate the impact a support group intervention had on the enhancement of self-worth of women who terminated a pregnancy in adolescence.^20,21^

### Study setting

The study was conducted in a CHC in Tshwane Metropolitan Municipality, South Africa. The CHC is one of the eight in Tshwane Health District, designated to run TOP and family planning clinics. The TOP clinic consults an average of 60 patients per month, according to clinic records. It serves predominantly African blacks in the neighbouring informal settlements and townships.

Activities of the support group (directed by the 1st researcher), were carried out at the CHC which serves the participants’ place of residence. All participants of the study were recruited from the same support group and were familiar with the CHC. The proximity of the CHC to both the researchers was also considered.

### Population and sampling

The population for this study was difficult to access from the outset because of the sensitivity and stigma related to the research topic. Using the purposive and snowball^19^ sampling techniques, seven participants were recruited. They each met the following inclusion criteria:

- terminated a pregnancy in adolescence
- 20–35 years old at the time of data collection
- were members of the same support group at the CHC, consisting of women who terminated a pregnancy by choice and experienced poor self-worth
- owning a smartphone, to form a chat group, for posting some of the presentations, for follow-up messaging by researchers, presenters or by participants, to affirm information and for the researchers’ need to clarify what was shared in the previous interviews
- willing to reflect and share in-depth, information-rich accounts of their experiences related to participation in the support group, which were needed to attain the study’s aim.^20^

The exclusion criteria were:

- unable to use a smart phone
- no mental, emotional or spiritual disturbance related to TOP
- older than 35 years
- member from a different support group
- not willing to share negative experiences related to TOP.

### Data collection

In-depth, individual, face to face interviews were conducted in a private office at the CHC in the Tshwane Metropolitan Municipality. Participants gave their permission for the researcher to audio record the interviews, which allowed her to concentrate on the interview. The interviews took place from 01 March 2022 to 31 March 2022 and lasted 45 min – 60 min each. Clinic rules to prevent the spread of coronavirus disease 2019 (COVID-19) were observed, including wearing masks, hand sanitising, keeping a 1.5 m distance during interviews, and ventilating the office.

**FIGURE 1:**
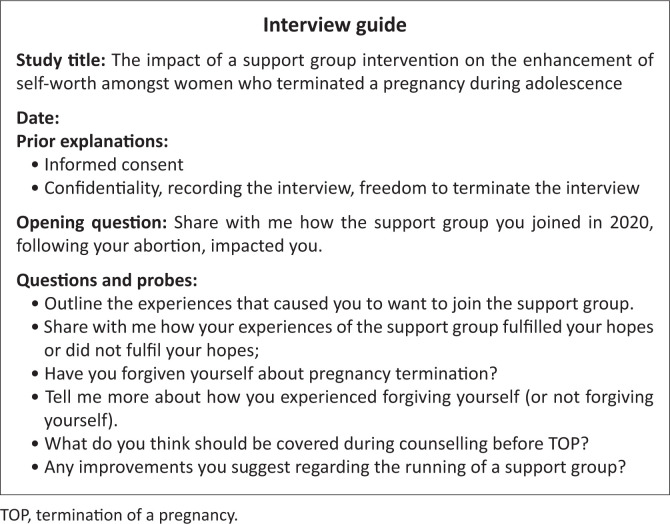
Interview guide.

Before each interview, the researcher explained the content of the consent form to the participant, had the participant sign the consent form, and explained the interview procedure. Although the consent form was written in English, participants were allowed to communicate in their preferred language. All participants preferred English. A broad opening question posed to participants was: ‘*Share with me how the support group you joined in 2020, following your abortion, impacted you*’.^21^ Participants were allowed to share their experiences in their own way. Subsequent probes were intended to prompt more detailed information. For consistency, an interview guide with a set of open-ended questions was used to guide, but not to dictate the interview (see Figure 1).^22^

Saturation was reached by the fifth participant, but the researcher nevertheless, included the remaining participants for a deeper understanding of the phenomenon.

### Data analysis

A six-phased thematic analysis approach guided the data analysis.^23^ The steps included:

- Becoming acquainted with the data by reading each transcript several times.
- Coding important features of the data relevant to the research objectives: An objective-based thematic structure was created and used to guide features of data, similar statements and content. These were organised into themes and plotted on a chart.
- Assembling coded data relevant to each theme: the researcher established similar and different emerging themes, and a logical structure was displayed.
- Reviewing themes, thereby ensuring they tell a convincing and absorbing story about the data.
- Describing and naming themes after identifying the essence of each: Basic themes were matched using sets of links to form organising themes. These were then classified under a global theme.
- Writing a coherent and persuasive story based on the findings and contextualise it in relation to current literature.^24^

## Results

### Demographic findings

The seven participants were women aged between 26 and 35 years. They all belonged to the Christian faith and were black Africans who terminated a pregnancy between the ages of 19 and 24 years, that is, during late adolescence.^25^ One had terminated the second pregnancy at age 29. Six participants had a child each, three were divorced at the time of the study whilst the rest (*n* = 4) were never married. Research affirms that teen pregnancy in developing countries mostly occurs outside wedlock.^26^ In some African communities, women are detested and isolated when discovered to have terminated a pregnancy.^27^ Communal knowledge that a girl aborted appears to reduce the woman’s value and her chances to get married.^27^ Even if TOP is legal in most countries, including South Africa, attitudes towards TOP remain unchanged.^28^

### Themes and sub-themes

Five themes and 12 sub-themes emerged from the data: (1) reasons for joining the support group with sub-themes, *confusion, stigma, guilt, not coping with thoughts about TOP and poor relationships;* (2) self-forgiveness, with sub-themes, *self-acceptance* and *accepting responsibility for TOP*; (3) enhanced emotional and physical wellbeing, with subthemes, *sharing problems within and outside the group and physical wellbeing*; (4) spiritual growth and (5) participants’ voices with sub-themes, *absenteeism in meetings, raising awareness about TOP,* and *pre-TOP counselling*.

### Reasons for joining the support group

The study was conducted 6 years after TOP for 1 participant, 9 years after TOP for 1 participant, 10 years after TOP for 2 participants, 11 years after TOP for 1 participant and 13 years after TOP for 2 participants.

Most women in the support group expressed they joined the group because of confusion, not coping with thoughts about TOP, poor relationships, and guilt about TOP, which later led to stress and depression. Religion has been theorised to position abortion stigma formation.^29^ Abortion stigma confuses a woman’s decision to terminate a pregnancy because of worries about judgement, isolation, self-judgement and community condemnation. The stigma of TOP motivated them to join the group because they carried a burden of secrecy and trusted no one outside the group. Participants described how they perceived that TOP taints or blemishes a woman’s character^27^ and classifies her as belonging to a type of ‘bad women’ who abandoned established norms of the community to which they belong.

Yet inside the group, they hoped to open up as they shared similar problems. However, one participant avoided joining the group earlier for fear of re-living her termination when other members shared their painful experiences.

### Confusion

Participant 1 shared:

‘Usually when a woman goes for abortion she is confused, you don’t know what you want. You end up taking wrong decisions. I needed to talk with someone about abortion … to understand what’s going on.’ (P1, 31 years old, employed)

### Stigma

As all participants in this study were Christians, they feared they would be judged severely by their spiritual friends, family or communities if their TOP was disclosed, hence the secrecy about TOP:

‘Abortion becomes a secret and the woman gets depressed, because she thinks she’s the only one who did it. With time, thinking about abortion will stress you.’ (P4, 35 years old, employed)

‘I feared sharing because I was not trusting anyone in the community … worried what people are going to say.’ (P1, 31 years old, employed)

### Guilt

Moreover, participants who reported greater levels of religiosity revealed feeling more self-condemning^30^:

‘It haunted me. It was heavy on me. I had guilt feelings about TOP, asking if God will ever forgive me.’ (P5, 32 years old, employed)

### Not coping with thoughts about termination of a pregnancy

One participant expressed she was still searching for the true meaning of terminating her pregnancy because she could not stop thinking about the experience. She shared:

‘I tell myself I can’t change anything, let me try to forget. I did not understand what I did. I kept reminding myself about TOP, hoping I may be “sharp”. I was not coping … It was like I did something bad, bad, bad, bad … so much remorse.’ (P6, 26 years old, employed)

### Poor relationships

In this study, three participants married after terminating their pregnancies and were later divorced, while one tolerated a relationship she defined as abusive.

Participant 4 shared about impatience and poor communication with family members:

‘At home I was emotionally not right with family. Not communicating right with them. I was impatient with family members.’ (P4, 35 years old, employed)

### Self-forgiveness

Self-acceptance and not blaming others for the termination were expressed by all participants as the outcome of forgiving oneself.

### Self-acceptance

All seven participants reported that they were able to forgive themselves after awareness about self-forgiveness was raised by one of the presenters. Participants found it easier to accept themselves:

‘It’s a phase I went through and I learnt from my mistakes. Let me leave the past where it is and use my energy to move on.’ (P1, 31 years old, employed)

‘I didn’t know about forgiving myself before, … I haven’t forgiven myself … I didn’t know about accepting myself as I am, with my mistakes … Through the teaching by the group leader on self-acceptance, that was posted on our smart phones, I learnt to forgive myself and others who advised me to abort because if you can’t forgive yourself you fail to forgive others and to get healing.’ (P5, 32 years old, employed)

### Accepting responsibility for termination of a pregnancy

Participants accepted they had shielded themselves from the responsibility of terminating their pregnancies, blaming their parents, the church, and their partners. For some, this culminated in dysfunctional relationships within the family or towards their partners. After a mobile phone teaching session on self-forgiveness and specifically on accepting responsibility and a discussion to acknowledge they were responsible for their actions,^12^ participants managed to stop blaming others who encouraged them to terminate their pregnancies. This reality encouraged participants to recognise the emotional harm the TOP caused them, as well as the emotional damage it could cause other children in the family. Sharing the negative emotions of self-hatred, self-punishment and self-condemnation with other women in the group made the internal resolution of these emotions easier.^12^ This was captured in the extract of two participants:

‘Accepting wrongdoing was not easy but having now accepted that I could now sleep at night without feeling any shame or guilt. I have found peace and I even apologised to my boyfriend for allowing him to impregnate me and for agreeing with him about the abortion.’ (P1, 31 years old, employed)

‘At home I was emotionally not right with family members. I was not communicating right with family members. I was impatient … full of hate to the people who asked me to terminate my pregnancy but now I know how to deal with those emotions.’ (P4, 35 years old, employed)

## Enhanced emotional and physical wellbeing

Most participants were relieved of the burden of secrecy, having managed to disclose and share with group members. According to some participants, they felt at ease asking for support and perceived this to be emotional strength and growth. Participants appreciated the teachings that were presented on divine forgiveness, self-forgiveness and assertiveness. They further reported that the skills they learnt would be useful in attaining some emotional relief.

### Sharing problems within and outside the group

It was a relief for participants to share about their reasons for TOP, having accepted and learned from their mistakes. Participants felt they did not need to hide their abortion experiences. They learnt this from the teachings about assertiveness and self-love.

This category is best captured by the following extracts:

‘We all openly shared about our wrongs that led to abortion … after that I got relieved. After my experiences within the support group I don’t need to hide my abortion. It is a part of myself. I learnt from teachings to be assertive … and to love myself. It encouraged me to be active, not afraid to learn from other women and visitors who came to teach us-so much so that I wish the group would never terminate, instead meet from Monday to Friday.’ (P3, 35 years old, unemployed)

‘I can talk to others outside the support group who aborted and have emotional problems, that they can continue to live according to their morals, that they must accept and … learn from their mistakes.’ (P2, 30 years old, unemployed)

### Physical wellbeing

Some participants reported that before they joined the group, looking clean and dressing well was not a priority for them. However, most felt they gained confidence and could now look after their physical needs and behave normally towards others; they realised it was up to them to be nice to themselves. One participant expressed:

‘… I know it is up to me to look beautiful for myself and not for anyone else and to believe in myself. I was angry for my ex-husband when he wanted another child after he forced me to abort the other one. I lived for him. I used to want to look beautiful for my ex-husband and not for me, but now I know I must be confident to do things for myself.’ (P7, 30 years old, employed)

### Spiritual growth

All the women understood God’s forgiveness because they belonged to the Christian faith. They all agreed that they experienced peace, developed spiritually and morally after involvement in the support group.^29^ They further expressed feeling spiritually healed, praying openly and feeling free to attend church and to help others who struggled with post-TOP self-hatred. In the light of such self-improvement, all the participants expressed a desire to share what caused them to cope.^31^

Three participants shared:

‘God forgave me because our God is a forgiving God. I am freed from the past. But I didn’t know, now I know. I’m fine and I moved on, although I actually did not forget everything. Sometimes I recall the experience … wish I should have done things differently.’ (P2, 30 years old, unemployed)

‘… joining a like- minded group you begin to realise you’re not alone. Once everyone shares their story you become free to talk. Even open up spiritually, like going to church, that is what I mean, yes.’ (P3, 35 years old, unemployed)

‘After joining support group, I am healed. I pray openly. I tell God He knows everything. I thank God that I met people who helped me open up.’ (P4, 35 years old, employed)

### Participants’ voices

The following are the miscellaneous sub-themes raised by participants, that is, absenteeism in meetings, raising awareness about TOP, feelings about group termination and counselling

#### Absenteeism in meetings

Absenteeism in support group meetings was attributed to family-related, transport-related, time-related factors, or the fear of regressing when members discussed aspects about the termination that some no longer wanted to hear. To prevent absenteeism, participants suggested that meetings be held on weekends. The following participants concurred:

‘Eish, I think maybe when I get to the meeting others in the group will say things about TOP that I don’t want to recall. It’s like I will “regress” again, think this and that. So I’m discouraged.’ (P6, 26 years old, employed)

‘… to generalise, some women have domestic problems …Problems can be at home or about transport, or time related.’ (P2, 30 years old, unemployed)

#### Raising awareness about termination of a pregnancy

Some participants emphasised the need for talks about TOP to be included as part of health education, for example alternatives to TOP or that the government should run TOP awareness campaigns:

‘Approach people in government that you formed a group, we achieved this and that. They benefited this or that. This should be implemented in every institution that has an abortion facility.’ (P1, 31 years old, employed)

‘We can form a group on Twitter and talk about our experiences on TOP. Then others can just join. There are never awareness campaigns about abortion or at talks the clinic.’ (P7, 30 years old, employed)

#### Pre-termination of a pregnancy counselling

Participant 6 emphasised the need for holistic counselling before TOP and that clinic managers should explain the reality of termination.

The truth about TOP should be shared before the client signs any documents, so they know and understand the consequences:

‘… what remains is that when I do it, I know I was told what I’m in for. I need to be told the experience will stay in my mind for long … It’s a person you were going to see, and then you just murdered him/her nje, nje, nje [*just, just, just*], just like that?’ (P6, 26 years old, employed)

Participant 6 further explained:

‘TOP is bad, but it is allowed … if the sister explained the reality about TOP, her voice is strong. So, when you do TOP you do it knowing the truth. It’s painful if you undergo it without knowing what you’re doing … that you have done a ‘bad’ thing does not appear in the paper we sign before TOP. I suggest this must appear in that paper. What will be the truth? The truth is: what will be done, emotions that will follow, that there are regrets, tell her its “mpe” [*bad*] but that it’s allowed. I believe if the TOP sister tells you, ‘you are doing such and such a thing’, and you agree to continue, it means you are ready, so that no one forced you.’ (P6, 26 years old, employed)

## Discussion

The purpose of the study was to explore and describe, from the women’s perspective, a support group intervention impact on the self-worth of women who terminated a pregnancy in adolescence. Although self-worth and self-esteem are sometimes used interchangeably, scholars define self-worth as a global assessment of oneself,^32,33^ whereas self-esteem is described as the extent to which an individual evaluates themselves favourably, based on perceived successes, achievements and being valued.^34^ In contrast to self-esteem, self-worth further entails a feeling that one deserves to be alive, loved and respected, as well as being of value and indispensable.^34^ Life satisfaction is related to a positive self-worth.^32^ The emotional pain stemming from a lack of self-worth following abortion can be the root of vulnerability to mental health challenges like depression, drug and alcohol misuse, among others.

### Reasons for joining the support group

Generally, participants joined the support group because they suffered the bondage of secrecy, guilt, confusion, stigma, not coping with thoughts about TOP and poor relationships which resulted in poor self-worth and self-condemnation. Causing harm to another person and to self can later cause sorrow, guilt, shame, self-blame and self-condemnation.^12^ In this study participants experienced abortion as an event through which they caused harm to themselves and to the foetus by violating their values. It is not necessary to perpetuate these feelings because restoration of self-worth through self-forgiveness can cause people to experience life satisfaction, self-trust, emotional stability, and positive relationships with others.^12^

#### Self-forgivenes

Genuine self-forgiveness is built on acceptance of one’s flawed nature and consequent inspiration to improve oneself.^12^ The process leading to genuine self-forgiveness involves accepting accountability for harm instigated on another and on the self. This notion is supported in the literature. It is claimed that when people accept responsibility for their role in TOP it causes them a deep, humbling sorrow, which enables them to deal effectively with their feelings, subsequently forgiving themselves genuinely and repenting for their actions.^12,29^ Sharing the negative emotions of self-hatred and self-condemnation with other women in the support group made the internal resolution of these emotions easier. This observation is supported by scholars,^35^ that, to discourage self-punishers from self-condemnation, they need a reassuring and safe social environment that supports their values.^35^ Participants in this study experienced the support group to be a safe setting where they freely shared their negative emotions. Feeling safe within the support group encouraged them to move from shame (causing the woman to condemn her whole self), to feelings of guilt (focusing on condemning the behaviour), henceforth no longer referring to themselves as murderers. They were able to move from shame to guilt and then to self-acceptance, which typically improved their self-worth.

Both accepting responsibility and expression of sorrow led to the desire to restore the values damaged by the decision to abort.^12,29^ This was done through reparative behaviours and recommitting to one’s values- values that were damaged by the decision to abort.^12^ Behaviour patterns that led to TOP, for example, lack of assertiveness were addressed. This paved the way for self-forgiveness. Finally, obtaining self-forgiveness and renewed self-compassion caused most participants to release negative emotions related to TOP. This did not mean forgetting that one acted wrongly but it served to remind them to avoid similar activities in the future. It also caused these women to recognise their worth as persons and approaching oneself with respect, compassion, acceptance and kindness.^12,35^.

After participants were taught about genuine self-forgiveness, they found they gained a new reality after accepting their liability for deciding to terminate their pregnancy.^36^

The intention to genuinely change, abandon self-resentment and diminish self-punishment, increase self-love and accept responsibility for terminating a pregnancy are important in enhancing self-acceptance.^37^

#### Enhanced emotional and physical wellbeing

The ultimate enhancement of emotional and physical wellbeing was another finding of this study. Research has shown that women who terminate a pregnancy suffer low self-esteem^38^ and anxiety.^39^ This was true of the participants in this study and it negatively affected their emotional and physical wellbeing. These feelings are attributed to a lack of social support as participants kept their shame and worry to themselves.^40^ The ability to verbalise and accept the termination helped enhance their emotional and physical wellbeing. Researchers assert that shamed women escape the painful feelings of shame by externalising blame on others, becoming self-centred, defensive and self-condemning.^41^ In contrast, women who feel guilty about their terminated pregnancy specifically condemn their behaviour with the intent to improve and stop hating themselves,^41^ although not necessarily getting rid of all feelings of regret.^12^ Although most women expressed having forgiven their ex-partners or family for encouraging them to terminate their pregnancies, they simultaneously revealed that the termination made them angry at those who coerced their decision. This possibility is supported by research, that when people forgive others, they may not get rid of all the negative feelings. Although forgiveness may entail decline of angry feelings, it does not necessarily require the cessation of emotions of sorrow, disappointment or regret.^35^

As one participant stated:

‘I was angry for my ex-husband when he wanted another child after he forced me to abort the other one. I lived for him … but now I know I must be confident to do things for myself.’ (P7, 30 years old, employed)

For some, this culminated in dysfunctional relationships within the family or towards their partners. Research shows dysfunctional relationships could be attributed to a woman’s anger towards her partner, who ignored her desire to keep the baby.^42^ It is stated that women who remain in abusive relationships become victims of violence, using their partners for self-punishment.^43^ It causes them to be less in touch with their emotions, resulting in outbursts of anger, which may be the only emotion they can truly feel. It is convenient for these women to remain in such relationships as they subconsciously believe this is the punishment they deserve, having aborted their foetal babies.^43^

That some women in this study were never married or were divorced could be related to their history of TOP. A study conducted among Ugandan men on their attitudes to abortion,^44^ highlights that these men believed if a married woman has an abortion, she must have been impregnated by another man and a husband could therefore be in conflict with such a situation. Abortion may help understand the cause of relationship problems, whether inside or outside marriage. A study on induced abortion and intimate relationship quality discovered that abortion experience within a current relationship was linked to increased risk of arguing about children of the couple, increased risk for sexual dysfunction among women and men being more likely to report jealousy.^42^ Research^45^ further reveals that such couples usually separate after TOP, owing to post-abortion distress which can increase the couple’s conflict, communication difficulty or destroy mutual trust, eventually leading to separation.^45^

#### Spiritual growth

Studies have found that religious affiliations shape negative attitudes about TOP, highlighting that pregnancy termination stigmatises and taints a woman’s character, classifying her as deviant from her established community norms.^30^ Participants in this study understood the difference between shame and guilt after it was explained (see Table 1). Although they did not forget everything about TOP and wished they should have performed things differently, some participants in this study felt freed and healed from the past guilt or self-condemnation. They believed their relationship with God and other Christians improved because God forgave them, they were free to pray and go to church to interact with other people. During the sessions on accepting accountability for aborting and expression of sorrow (self-forgiveness session), all participants were encouraged to accept responsibility for TOP, without blaming anyone.^12^ They were further encouraged to point out the consequences of TOP and the harm caused on themselves and the foetus.

Participants needed to review the factors that contributed to unwanted or unplanned pregnancy and TOP.

This would cause participants to understand what caused them to act the way they did.^12^ At the end of the accountability and sorrow session, participants were aware that continuing self-condemning themselves served no purpose. Understanding the difference between shame and guilt helped participants realise that bad actions do not necessitate a bad person identity, thereby reducing their shame.^12^ Approaching oneself with compassion reduces shame. Research reveals that moral and spiritual developments are socially valued outcomes which generate pride, consequently enhancing self-worth.^41^ It was proper for participants in this study to reaffirm their values that have been disrupted by TOP and thereafter to release their negative emotions after forgiving themselves. Failure to achieve self-forgiveness would serve no useful purpose.^12^

Although these women stated to never forget their aborted babies and the trauma of termination, they accepted and made peace with themselves. They felt emotionally resilient to stand their ground when facing challenges in life, but acknowledged they would always wish they could have acted differently. The discussion sessions within the group enabled participants to reflect on their past behaviour and decide to move on. All participants shared that they experienced a decreased sense of stigma, felt better control over their lives, gained better social skills within their families, and felt relief from guilt. These lifelong skills left them feeling empowered with enhanced self-worth.

#### Participants’ voices

The central sub-theme that emerged emphasised that counselling before TOP by the clinic manager should explain the reality of pregnancy termination so that they know and understand the consequences of abortion before signing.

Research indicates that most of the pre-TOP information addresses physical risks,^46^ excluding termination of the developing fetus, the possibility of regret, the need for support and the negative emotions that may stay long in the woman’s mind. Hence, many women undergo abortion before fully understanding its repercussions. Feeling misinformed prior TOP may lead to post-abortion difficulties.^46^ Accurate information about foetal development ensures women make a decision consistent with their beliefs and value systems. Using terms such as ‘blob of cells’ when referring to a 6-week fetus is regarded by some health care providers as simplifying the decision-making. Consistent with participant 6’s narrative, others may find it dishonest because for decision making, women deserve to know what would be removed. Protecting them from the facts deprives them of informed consent.^46^

Miscellaneous sub-themes that arose were about non-participation in meetings because of absenteeism, which caused failure of participants to fully experience the emotional and informational benefits^31^ of the support group. Participants suggested meetings should be held on weekends.

Silence around the topic of abortion as part of the regular health education presented to patients at clinics was also noted. It is recommended that the government runs TOP awareness campaigns in the media, like it does with human immunodeficiency virus (HIV) and acquired immunodeficiency syndrome (AIDS).

### Limitation of the study

One of the limitations is that the sample was too small to generalise the findings. Furthermore, the sample was homogenous, consisting of individuals identifying as black and Christian, and were from a low socioeconomic background. Future research based on a more diverse sample regarding race and religious diversity, could produce different results after assessing replicability and generalisability.

Data were collected on average, 9 years after TOP yet, the study relied on participants’ recall. Previous research has revealed issues with retrospective memory that individuals tend to recall events in a self-enhancing manner.^47^ It is possible that participants could have been biased in recalling their experiences about TOP.

### Recommendations

It is recommended that a support group should be considered as a backup for women who terminated a pregnancy and have lost their self-worth. The core intervention for women who lost their self-worth is to give them unconditional love and respect inside and outside the support group.^8^ Furthermore, women should be encouraged to accept their own self-worth, after raising awareness on the difference between self-worth and self-esteem.^48^ Self-esteem is what people think, sense and believe about themselves, whereas self-worth is a deep knowing that we are of value, loveable, essential to this life, and of inexplicable worth, despite our failures.^8^

Owing to controversy about the negative mental health outcomes associated with this phenomenon, there is a need for further research to improve screening and counselling to reduce the adverse outcomes of terminating a pregnancy.^5^

## Conclusion

The study’s findings revealed that participation in a support group is effective in promoting self-worth of women who terminated a pregnancy. It was not possible to assess the intervention impact over the long term, but it was clear that the intervention was effective in the short term as it did improve participants’ self-worth by altering their self-condemning emotions, replacing them with self-respect and self-love.
